# Assessment of Lifestyle Factors Helps to Identify Liver Fibrosis Due to Non-Alcoholic Fatty Liver Disease in Obesity

**DOI:** 10.3390/nu13010169

**Published:** 2021-01-08

**Authors:** Mari Lahelma, Panu K. Luukkonen, Sami Qadri, Noora Ahlholm, Susanna Lallukka-Brück, Kimmo Porthan, Anne Juuti, Henna Sammalkorpi, Anne K. Penttilä, Johanna Arola, Marju Orho-Melander, Hannele Yki-Järvinen

**Affiliations:** 1 Minerva Foundation Institute for Medical Research, 00290 Helsinki, Finland; mari.lahelma@helsinki.fi (M.L.); panu.luukkonen@yale.edu (P.K.L.); sami.qadri@helsinki.fi (S.Q.); noora.ahlholm@fimnet.fi (N.A.); susanna.lallukka-bruck@helsinki.fi (S.L.-B.); kimmo.porthan@fimnet.fi (K.P.); 2 Department of Medicine, University of Helsinki, Helsinki University Hospital, 00290 Helsinki, Finland; 3 Department of Internal Medicine, Yale University School of Medicine, New Haven, CT 06520-8056, USA; 4 Abdominal Center, Department of Gastrointestinal Surgery, Helsinki University Hospital, 00290 Helsinki, Finland; anne.juuti@hus.fi (A.J.); henna.sammalkorpi@hus.fi (H.S.); anne.k.penttila@hus.fi (A.K.P.); 5 Department of Pathology, Helsinki University Hospital, University of Helsinki, 00290 Helsinki, Finland; johanna.arola@hus.fi; 6 Diabetes and Cardiovascular Disease-Genetic Epidemiology, Department of Clinical Sciences in Malmö, Lund University, 20502 Malmö, Sweden; marju.orho-melander@med.lu.se

**Keywords:** physical activity, diet, stress, glucose, insulin

## Abstract

Only some individuals with obesity develop liver fibrosis due to non-alcoholic fatty liver disease (NAFLD-fibrosis). We determined whether detailed assessment of lifestyle factors in addition to physical, biochemical and genetic factors helps in identification of these patients. A total of 100 patients with obesity (mean BMI 40.0 ± 0.6 kg/m^2^) referred for bariatric surgery at the Helsinki University Hospital underwent a liver biopsy to evaluate liver histology. Physical activity was determined by accelerometer recordings and by the Modifiable Activity Questionnaire, diet by the FINRISK Food Frequency Questionnaire, and other lifestyle factors, such as sleep patterns and smoking, by face-to-face interviews. Physical and biochemical parameters and genetic risk score (GRS based on variants in *PNPLA3*, *TM6SF2*, *MBOAT7* and *HSD17B13*) were measured. Of all participants 49% had NAFLD-fibrosis. Independent predictors of NAFLD-fibrosis were low moderate-to-vigorous physical activity, high red meat intake, low carbohydrate intake, smoking, HbA_1c_, triglycerides and GRS. A model including these factors (areas under the receiver operating characteristics curve (AUROC) 0.90 (95% CI 0.84–0.96)) identified NAFLD-fibrosis significantly more accurately than a model including all but lifestyle factors (AUROC 0.82 (95% CI 0.73–0.91)) or models including lifestyle, physical and biochemical, or genetic factors alone. Assessment of lifestyle parameters in addition to physical, biochemical and genetic factors helps to identify obese patients with NAFLD-fibrosis.

## 1. Introduction

Non-alcoholic fatty liver disease (NAFLD) covers a spectrum from simple steatosis (NAFL) to non-alcoholic steatohepatitis (NASH) and various stages (F1–F4) of fibrosis [1]. Fibrosis is the best predictor of mortality in NAFLD [2,3]. The prevalence of liver fibrosis due to NAFLD (NAFLD-fibrosis) averages 40% (≥F1, range 13–97%) in patients with morbid obesity [4]. The reason for this interindividual variation is poorly understood, as data on predictors of histologically determined NAFLD-fibrosis are sparse.

Previous studies have mainly explored associations between physical factors, such as measures of body weight and composition, and biochemical parameters including features of the metabolic syndrome, and NAFLD-fibrosis [5,6,7,8]. The possibility that assessment of several lifestyle-related factors such as diet, physical activity, fatigue and stress and sleep patterns in addition to physical and biochemical features and common powerful genetic variants (vide infra) help in predicting biopsy-proven NAFLD-fibrosis has not been explored. There are some data on single lifestyle factors and their relationship to NAFLD-fibrosis. Adherence to a Mediterranean diet was significantly less frequent in those with NAFLD-fibrosis compared to those without in a cross-sectional study of 82 patients with obesity [9], and high daily fructose consumption was found to be significantly associated with liver fibrosis in another cross-sectional study [10]. Self-reported physical activity (PA) has been found to associate inversely with fibrosis stage in NAFLD [11], but there are no data relating objectively quantified PA to NAFLD-fibrosis. Regarding other lifestyle factors, daytime sleepiness was positively correlated with the degree of fibrosis in a cross-sectional study of 46 patients with biopsy-proven NAFLD and 22 healthy controls [12]. Advanced NAFLD-fibrosis may also be more common in smokers than in non-smokers [13].

Common genetic polymorphisms in patatin-like phospholipase domain-containing 3 (*PNPLA3*) at rs738409, transmembrane 6 superfamily member 2 (*TM6SF2*) at rs58542926, membrane-bound O-acyltransferase domain-containing 7 (*MBOAT7*) at rs641738 and 17-beta-hydroxysteroid dehydrogenase (*HSD17B13*) at rs72613567 have been shown to robustly influence the risk of NAFLD-fibrosis [14]. Polygenic risk scores have emerged as a powerful tool in studying the compound effect of multiple genetic variants on phenotype in multifactorial diseases [15]. Recently, Gellert-Kristensen et al. showed that a genetic risk score (GRS) incorporating the NAFLD risk-modifying alleles in *PNPLA3, TM6SF2* and *HSD17B13* was significantly associated with cirrhosis [16]. Whether GRS helps in diagnosing NAFLD-fibrosis in patients with obesity, whose physical and biochemical and lifestyle parameters have been assessed, is unknown.

**Figure 1:**
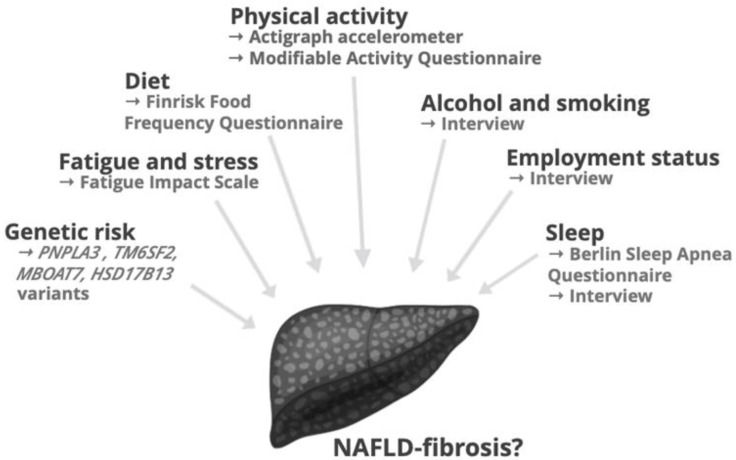
An overview of lifestyle and genetic factors investigated in the study. Abbreviations: *HSD17B1*, 17-β hydroxysteroid dehydrogenase; *MBOAT7*, membrane bound O-acyltransferase domain containing 7; NAFLD, non-alcoholic fatty liver disease; *PNPLA3*, patatin-like phospholipase domain-containing protein 3; *TM6SF2*, transmembrane 6 superfamily member 2.

Thus, in the present study, we were interested in determining whether simultaneous assessment of multiple lifestyle factors including diet, objectively measured physical activity, fatigue and stress, sleep patterns, employment status and smoking, in addition to physical, biochemical and genetic factors, helps in identification of NAFLD-fibrosis. This would seem valuable, as assessment of lifestyle habits is inexpensive and important for educational and therapeutic purposes in the clinic. For this, we comprehensively characterized these factors as schematically depicted in Figure 1 in 100 obese patients who underwent a liver biopsy.

## 2. Materials and Methods

### 2.1. Participants

We prospectively recruited 100 consecutive participants amongst those referred for bariatric surgery at the Helsinki University Hospital (Helsinki, Finland). The participants were eligible if they met the following inclusion criteria: (a) age 18 to 75 years; (b) no known acute or chronic disease except for obesity, type 2 diabetes or hypertension on the basis of medical history, physical examination and standard laboratory tests (complete blood count, serum creatinine, C-reactive protein, thyroid stimulating hormone and electrolyte concentrations); (c) alcohol consumption less than 20 g per day for women and less than 30 g per day for men; (d) no evidence of liver conditions other than NAFLD such as autoimmune, drug-induced or viral liver disease; (e) no history of use of toxins or drugs associated with liver steatosis; (f) not pregnant or lactating.

The study protocol was approved by the ethics committee of the Hospital District of Helsinki and Uusimaa (78/13/03/01/2015), and it was performed in accordance with the Declaration of Helsinki. Each participant provided their written informed consent after being explained the nature and potential risks of the study.

### 2.2. Study Design

Prior to bariatric surgery, the participants were invited to two separate visits to the Clinical Research Unit for comprehensive characterization of lifestyle, physical and biochemical, and genetic factors.

At the first visit, approximately two weeks prior to surgery, informed consent was obtained, body height and weight were recorded, and a tri-axial accelerometer was provided to be worn for the following seven days, as detailed below. At the second visit, approximately one week prior to surgery, blood samples were taken for laboratory tests and genotyping, as detailed below. Physical measurements and face-to-face interviews assessing medical history, duration of obesity, smoking habits, diet (FINRISK Food Frequency Questionnaire), PA (Modifiable Activity Questionnaire), fatigue (Fatigue Impact Scale) and sleep (Berlin Sleep Apnea Questionnaire) were performed, and the accelerometer data were analyzed.

### 2.3. Lifestyle Factors

#### 2.3.1. Physical Activity

(i) Accelerometer recordings. At the first clinical visit, the participants were provided with a tri-axial accelerometer (ActiGraph wGT3X-BT, Actigraph LLC, Pensacola, FL, USA) to be worn on their right hip during waking hours except water-based activities (e.g., showering) for the following seven days, while encouraged to maintain their normal PA. The device measures body position (lying/sitting/standing) using an inclinometer. Raw data were analyzed using Actilife 6.0 (Actigraph Software Department, Pensacola, FL, USA). Cut points by Freedson Adult VM (2011) were used to convert raw accelerations as counts per minute to PA intensities [17]. PA intensities were defined as: sedentary behavior (<1.5 metabolic equivalents of task (METs)), light PA (1.5–3.0 METs), moderate PA (3.1–6.0 METs) and vigorous PA (>6.0 METs). Only participants with recordings of ≥9 h per day for ≥4 days were included to further analysis.

(ii) Modifiable Activity Questionnaire. In addition to accelerometer recordings, PA was assessed using the Modifiable Activity Questionnaire (MAQ), translated into Finnish [18]. The questionnaire has been validated in several cohorts and shown to predict type 2 diabetes [18,19,20]. MAQ assesses in detail leisure and occupational PA as well as sedentary behavior during the past 12 months. The average weekly energy expenditure (EE) of PA and time spent in moderate-to-vigorous physical activity (MVPA) were calculated.

#### 2.3.2. Diet

The FINRISK Food Frequency Questionnaire (FFQ) was used to characterize the diet of the participants [21]. The FINRISK FFQ has been developed by the Finnish Institute for Health and Welfare to assess dietary patterns in Finns. It examines monthly, weekly and daily intake of 56 food items, in addition to meal frequency, type and quality of selected food items, and alcohol consumption. Food items were categorized as follows: grains (e.g., biscuits, pizza, pasta, porridge), dairy products (e.g., yoghurt, cheese, ice cream), potatoes and vegetables (e.g., steamed potato, french fries, salad), fruits and berries, fish (e.g., salmon, Baltic herring), meat (e.g., red meat, poultry, cold cuts, eggs) and others (e.g., chocolate, snacks, convenience foods) Food intake frequencies were quantified as: never = 1, few portions in a month = 2, one portion a week = 3, two to four portions a week = 4, five to six portions a week = 5, daily = 6. Nutritional composition and energy percentages (E%) of macronutrients were determined by entering the FFQ data into the National Food Composition Database of Finland [22]. Energy densities of macronutrients were 4 kcal/g for carbohydrates, 4 kcal/g for proteins and 9 kcal/g for fats.

#### 2.3.3. Sleep, Stress and Socioeconomic Factors

The Fatigue Impact Scale was used to assess fatigue symptoms in the participants [23]. The scale has been previously used for measuring fatigue amongst patients with NAFLD and obesity [24,25]. The questionnaire assesses fatigue symptoms during the previous month with 40 items divided into three subdomains: cognitive (10 items), physical (10 items) and psychosocial (20 items) [23].

The risk of sleep apnea was assessed using the Berlin Sleep Apnea Questionnaire, which has been developed to identify individuals at a high risk for obstructive sleep apnea in primary care [26]. The questionnaire consists of three domains and classifies patient as having either a high or a low risk for sleep apnea.

Sleeping habits, smoking status, employment, duration of overweight, and medical history, were assessed during the face-to-face interview.

### 2.4. Physical and Biochemical Factors, and Liver Histology

Activities of alanine aminotransferase (ALT), aspartate aminotransferase (AST), glutamyl-transferase (GGT) and concentrations of high-density lipoprotein (HDL) cholesterol and low-density lipoprotein (LDL) cholesterol, triglycerides, glucose, glycosylated hemoglobin A_1c_ (HbA_1c_), insulin, albumin and platelet count were measured as described previously in detail [27]. The homeostasis model assessment of insulin resistance (HOMA-IR) was calculated using the formula: fasting glucose (mmol/L) × fasting insulin (mU/L)/22.5 [28]. Blood pressure as well as body weight, height, and hip and waist circumferences, and waist-to-hip ratio (WHR), were measured as described in [29].

Wedge biopsies of the liver were obtained at the beginning of bariatric surgery. Liver histology was analyzed by an experienced liver pathologist (J.A.) in a blinded fashion. Fibrosis stage was reported as proposed by Brunt et al. [1] as: F0, absence of fibrosis; F1, perisinusoidal or periportal fibrosis; F2, portal/periportal fibrosis; F3, bridging fibrosis; and F4, cirrhosis. NAFLD-fibrosis was defined as F1-F4 and absence of fibrosis as F0.

### 2.5. Genetic Factors

Genotyping of *PNPLA3* at rs738409, *TM6SF2* at rs58542926, *MBOAT7* at rs641738 and *HSD17B13* at rs72613567 was performed as previously described [30,31,32]. To investigate the impact of all four single-nucleotide polymorphisms (SNPs) on NAFLD-fibrosis, a GRS was calculated as suggested [16] by summing up the number of minor risk alleles (0–2) in *PNPLA3*, *TM6SF2* and *MBOAT7*, as well as major alleles (0–2) in *HSD17B13*. Thus, GRS ranged from 0 to 8 [4].

### 2.6. Statistical Analyses

The D’Agostino-Pearson omnibus normality test was used to analyze the distribution of continuous variables. Normally distributed data are shown as mean ± standard error of mean (SEM) and non-normally distributed data as median followed by the 25th and 75th percentiles. The unpaired Student’s *t* test and Mann-Whitney U test were used to compare normally and non-normally distributed groups, respectively. Categorical data are shown as proportions and compared using the Fisher’s exact test or the Chi-square test, as appropriate.

For each variable, univariable logistic regression analysis was used to calculate odds ratio (OR) and 95% confidence interval (CI) for NAFLD-fibrosis. Variables associated with NAFLD-fibrosis at a significance level of ≤0.05 in univariable analysis were included in the multivariable logistic regression using stepwise backward selection with 0.10 probability for stepwise removal. If the variables measured essentially the same biological phenomenon such as glucose/HbA_1c_, HOMA-IR/insulin, carbohydrate/protein/fat energy percent [E%]), only the variable that was most significantly associated with NAFLD-fibrosis was included. Five models were built by including in the analysis either lifestyle (1), physical and biochemical (2), or genetic factors (3) alone, all but lifestyle factors (4), and finally, all the factors (5). To measure abilities of the models to separate participants with and without liver fibrosis, areas under the receiver operating characteristics curves (AUROCs) were calculated [33]. The differences between AUROCs of the models were compared using the method described by DeLong et al. [34].

## 3. Results

A total of 94 (94%) participants completed the study. Six participants were excluded due to excessive alcohol intake, coagulation disorder and last-minute refusal to undergo bariatric surgery. A total of 49% of the participants had histologically determined NAFLD-fibrosis. Lifestyle characteristics of the participants with (FIB+) and without (FIB−) NAFLD-fibrosis are shown in Table 1 and physical, biochemical, histological and genetic characteristics in Table 2.

### 3.1. Lifestyle Factors

#### 3.1.1. Physical Activity

(i) Accelerometer recordings. The time spent in light PA was associated with a significantly decreased risk and sedentary behavior with a significantly increased risk of NAFLD-fibrosis (Table 1).

(ii) Modifiable Activity Questionnaire. The EE of occupational PA and time spent in MVPA were significantly lower amongst participants in the FIB+ than in the FIB- group, and in univariable analysis time spent in MVPA was also significantly associated with a decreased risk of NAFLD-fibrosis (Table 1).

#### 3.1.2. Diet

In all participants, 41 (37–46) E% of total calories was derived from carbohydrate, 19 (18–23) E% from protein and 35 (31–39) E% from fat. The energy derived from carbohydrates was significantly lower, while that from fats, especially from saturated fatty acids (SFA), was significantly higher in the FIB+ compared to the FIB− group. The participants in the FIB+ group consumed more red meat than the participants in the FIB− group (Table 1). In univariable analysis, red meat intake and E% from fats were associated with a significantly increased risk of NAFLD-fibrosis and E% from carbohydrates with a significantly decreased risk of NAFLD-fibrosis (Table 1).

#### 3.1.3. Sleep, Stress and Socioeconomic Factors

Participants in the FIB+ group consumed significantly more alcohol than participants in the FIB- group, although alcohol consumption was within the inclusion criteria for NAFLD in all participants (<20 g alcohol/day for women and <30 g alcohol/day for men) (Table 1). The proportion of participants who were current or former smokers or unemployed was higher in the FIB+ than the FIB− group (Table 1).

### 3.2. Physical and Biochemical Factors, and Liver Histology

The median age of participants was 51 (44–57) years and BMI averaged 40.0 ± 0.6 kg/m^2^. The participants in the FIB+ as compared to the FIB- group had significantly higher WHR, HbA_1c_, insulin, HOMA-IR, GGT and triglycerides (Table 2). They also had an increased prevalence of the metabolic syndrome (MetS), type 2 diabetes and glucose-lowering medication, and were more often men (Table 2).

All of the histologic features of NAFLD (macro-vesicular steatosis, ballooning, inflammation and grade of activity) were significantly more prevalent in the FIB+ than in the FIB− group (Table 2).

### 3.3. Genetic Factors

The participants in the FIB+ group were more often carriers of at least one risk variant allele in *PNPLA3* at rs738409 and had a higher GRS than those in the FIB− group (Table 2).

### 3.4. Multivariable Analyses

Models including lifestyle information alone (‘Lifestyle model’), physical and biochemical features alone (‘Biomarker model’), GRS alone (‘Genetic model’), both physical and biochemical parameters and GRS (‘Biomarker + Genetic model’) and all of these (‘Comprehensive model’) were built.

**Figure 2:**
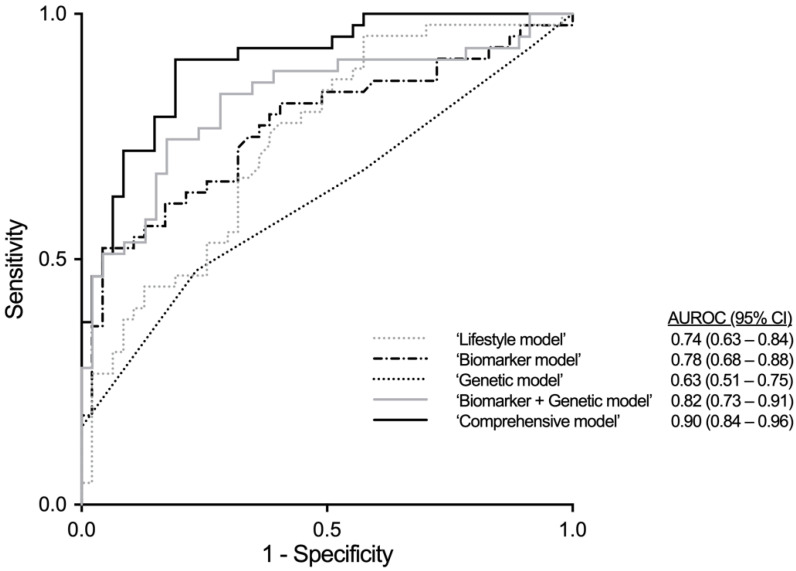
Receiver operating characteristic (ROC) curves of the multivariable models to predict NAFLD-fibrosis (F ≥ 1). The areas under ROC curves (AUROC) were 0.74 for the ‘Lifestyle model’ (including red meat intake, carbohydrate E% and unemployment), 0.78 for the ‘Biomarker model’ (including HbA_1c_, insulin and triglycerides), 0.63 for the ‘Genetic model’ (including genetic risk score), 0.82 for the ‘Biomarker + Genetic model’ (including HbA_1c_, insulin, triglycerides and genetic risk score) and 0.90 for the ‘Comprehensive model’ (including HbA_1c_, triglycerides, moderate-to-vigorous physical activity time, red meat intake, carbohydrate E%, smoking and genetic risk score). Abbreviations: AUROC, area under receiver operating characteristic curve; CI, confidence interval.

We included lifestyle variables that significantly associated with fibrosis in univariable analysis (Table 1) (light PA and sedentary behavior (accelerometer), time spent in MVPA (MAQ), carbohydrate E%, red meat intake, smoking, unemployment) in the ‘Lifestyle model’. The remaining independent predictors of NAFLD-fibrosis in this model were red meat intake, carbohydrate E% and unemployment (Table 3). The AUROC of the ‘Lifestyle model’ was 0.74 (95% CI 0.64–0.84) (Figure 2).

Physical and biochemical variables that significantly associated with fibrosis in univariable analysis (Table 2) were included in the ‘Biomarker model’ (sex, WHR, HbA_1c_, insulin, triglycerides, MetS, type 2 diabetes). The independent remaining predictors of NAFLD-fibrosis were HbA_1c_, insulin and triglycerides (Table 3). The AUROC of the ‘Biomarker model’ was 0.78 (95% CI 0.68–0.88) (Figure 2).

For the ‘Genetic model’, GRS in addition to sex was included, and GRS predicted fibrosis independent of sex (Table 3). The AUROC of the ‘Genetic model’ was 0.63 (95% CI 0.51–0.75) (Figure 2). Because of individual genetic variants *PNPLA3* was the only one with significant association to liver fibrosis in univariate analysis, so we also measured performance of the model using only this variant instead of GRS. As the model with GRS predicted liver fibrosis with higher sensitivity and specificity as measured by AUROCs (data not shown), we included it to the multivariable models.

For the ‘Biomarker + Genetic model’, sex, WHR, HbA_1c_, insulin, triglycerides, MetS, type 2 diabetes and GRS were included. The remaining predictors were HbA_1c_, insulin, triglycerides and GRS. The AUROC of the ‘Biomarker + Genetic model’ was 0.82 (95% CI 0.73–0.91) (Figure 2).

Finally, all significant predictors from univariable analysis (Table 1 and Table 2) were included in the ‘Comprehensive model’. The remaining predictors were time spent in MVPA (MAQ), red meat intake, carbohydrate E%, smoking, HbA_1c_, triglycerides, and GRS (Table 3). The AUROC of the ‘Comprehensive model’ was 0.90 (95% CI 0.84–0.96) (Figure 2).

The AUROC of the ‘Comprehensive model’ was significantly better than the AUROC of the ‘Lifestyle model’ (*p* = 0.002 for comparison between models), ‘Biomarker model’ (*p* = 0.011), ‘Genetic model’ (*p* < 0.001) or ‘Biomarker + Genetic model’ (*p* = 0.043).

## 4. Discussion

Fibrosis is the best predictor of mortality in NAFLD [2,3]. Identification of patients especially at early stages of fibrosis is important as disease regression is possible by weight loss [35]. Since mild fibrosis can only be reliably quantified by using an invasive liver biopsy, diagnosis of early NAFLD-fibrosis is challenging. In the present study, independent predictors of NAFLD-fibrosis amongst obese patients referred for bariatric surgery were low self-reported moderate-to-vigorous PA, high red meat and low carbohydrate intake, current or former smoking, increased HbA_1c_ and serum triglycerides and high GRS. We found that assessment of lifestyle factors in addition to the usual physical and biochemical predictors and GRS significantly helps in predicting NAFLD-fibrosis.

These data are the first to analyze the relationship between objectively measured PA and biopsy-proven NAFLD-fibrosis in individuals with obesity. Light intensity PA (1.5–3.0 METs) was inversely and sedentary behavior (<1.5 METs) positively associated with NAFLD-fibrosis. Light intensity PA includes activities of daily living such as casual walking or household chores, while sedentary behavior comprises activities like driving or doing desk work at a computer. These activities are rarely reported in PA questionnaires but can be derived from portable accelerometer data. In addition to accelerometer recordings, the questionnaire data showed that self-reported time spent in moderate-to-vigorous PA was inversely related to NAFLD-fibrosis and subject to large interindividual variation (25th–75th percentile 2.7–20.8 h/week). We hypothesize based on the current study that not only moderate-to-vigorous PA, but also light intensity PA, might be helpful in preventing NAFLD-fibrosis in patients with obesity. It would be important to test this hypothesis in a randomized clinical trial.

Of the dietary parameters, high relative intake of fat, especially saturated fat, in addition to high red meat intake, low relative intake of carbohydrate and high alcohol consumption, were significantly associated with NAFLD-fibrosis. Of these variables, red meat intake showed the strongest association. This finding is in line with previous data showing red meat to increase the risk of chronic liver disease [36]. The mechanisms linking intake of red meat to advanced liver disease are uncertain but could be related to saturated fat in red meat [37]. Regarding alcohol, the results of the present study are consistent with recent prospective data from Finland, which showed that the risk of severe liver disease increases slightly but steadily at all levels of alcohol consumption, even within the range deemed acceptable for the diagnosis of NAFLD (<20 g/day for women and <30 g/day for men) [38].

Regarding physical and biochemical factors, the frequently assessed waist-to-hip ratio and plasma glucose, insulin, GGT, and triglycerides were significant predictors of NAFLD-fibrosis, in keeping with previous data [7,39]. In addition, GRS significantly associated with NAFLD-fibrosis, consistent with data showing that GRS predicts risk of cirrhosis in the general population [16] and NASH amongst Korean individuals with biopsy-proven NAFLD [40]. Laboratory tests for these gene variants, other than a test for the *PNPLA3* rs738409 variant in some academic centers, are not routinely available in clinical practice. In the current cohort, AUROC of GRS alone was poor compared to other models implying that merely genotyping of the variants included in GRS is not sufficient to diagnose NAFLD-fibrosis.

The participants in the current study were obese and referred for bariatric surgery. This is an appropriate cohort if the goal is to search for predictors of liver fibrosis in the setting of an obesity clinic, but the data do not necessarily apply to mildly overweight or normal-weight individuals. As the study also enrolled participants in the greater area of Helsinki, the results may not apply to individuals from other regions of the world. The present cohort is large compared to previous studies searching for markers of histologically verified NAFLD-fibrosis [41,42,43], but the results should be considered as hypothesis-generating and need further verification. This is a challenge, however, as a liver biopsy is needed particularly in obese subjects to accurately and successfully assess liver fibrosis.

## 5. Conclusions

The present data show that amongst obese individuals eligible for bariatric surgery, those who are physically inactive, consume red meat, smoke, have elevated HbA_1c_ and triglycerides, and who are genetically predisposed based on GRS, are the ones susceptible for NAFLD-fibrosis. Thus, the same lifestyle factors which identify those at risk for cardiovascular disease may also increase the risk of NAFLD-fibrosis [44,45,46]. These data emphasize the importance of assessing dietary and physical habits and smoking for predicting NAFLD-fibrosis and raise the possibility that development of NAFLD-fibrosis could be prevented by treatment of these modifiable risk factors.

## Figures and Tables

**Table 1:** Lifestyle characteristics of the participants without (FIB−) and with (FIB+) NAFLD-fibrosis, and univariable analysis to predict NAFLD-fibrosis.

	NAFLD-Fibrosis		OR (95% CI)
	No (FIB−, *n* = 48)	Yes (FIB+, *n* = 46)	
Physical activity			
Accelerometer			
Use (h/day)	12.4 (11.3–13.9)	11.7 (10.6–13.4)	0.94 (0.74–1.19)
Steps (per day)	4981 (3775–6503)	4872 (4062–6628)	0.84 (1.00–1.01)
PA EE (MET-h/week)	30 (21–43)	30 (19–42)	1.00 (0.98–1.02)
MVPA (h/week)	2.1 (0.9–3.7)	2.0 (0.8–3.7)	1.00 (0.88–1.19)
Light PA (%)	32 (27–37)	31 (25–34)	0.94 (0.89–0.99) *
Sedentary (%)	65 (57–70)	68 (62–73)	1.06 (1.01–1.11) *
Sitting (h/day)	6.6 ± 0.2	6.6 ± 0.2	1.01 (0.77–1.33)
Modifiable Activity Questionnaire			
PA EE (MET-h/week)	36 (17–128)	27 (9–77)	0.97 (0.93–1.00)
Occupational PA (MET-h/week)	3 (0–87)	0 (0–12) *	0.99 (0.98–1.00)
Leisure time PA (MET-h/week)	20 (12–65)	18 (9–32)	1.00 (0.98–1.03)
MVPA (h/week)	8.1 (4.4–31.4)	5.7 (2.0–16.5) *	0.97 (0.93–0.99) *
Screen time (h/day)	3 (2–4)	4 (3–6)	1.16 (0.98–1.37)
Diet			
Carbohydrates E%	43 ± 1	39 ± 1 *	0.91 (0.85–0.98) *
Proteins E%	19 (18–21)	20 (19–23)	1.12 (0.99–1.27)
Fats E%	33 (28–38)	36 (33–40) *	1.09 (1.01–1.17) *
MUFA E%	10.7 ± 0.5	11.5 ± 0.4	1.10 (0.96–1.27)
PUFA E%	5.5 ± 0.2	5.6 ± 0.2	1.03 (0.79–1.34)
SFA E%	10.3 ± 0.3	11.3 ± 0.4 *	1.16 (0.98–1.37)
Fiber (g/day)	21 (16–31)	19 (14–29)	0.98 (0.94–1.02)
Salt (g/day)	6.0 ± 0.3	5.7 ± 0.3	1.01 (0.85–1.19)
Coffee (cups/day)	3 (1–5)	3 (1–5)	1.00 (0.98–1.02)
Fruits & Vegetables ^#^	4.6 ± 0.2	4.3 ± 0.2	0.93 (0.78–1.12)
Red meat ^#^	1 (1–3)	3 (1–3) *	1.76 (1.18–2.64) **
Sleep, stress and socioeconomic factors			
Sleep length (h)	7.5 (6.7–8.0)	8.0 (6.8–8.5)	1.13 (0.80–1.60)
Sleep apnea (yes)	13 (28%)	18 (40%)	1.74 (0.73–4.18)
Fatigue ^&^	19 (9–31)	27 (14–42)	1.01 (0.99–1.03)
Alcohol (portions/week) ^##^	0.6 ± 0.4	1.5 ± 0.4 **	1.16 (0.95–1.41)
Smoking (never/current/former)	24/4/19	11/4/31 **	3.71 (1.49–9.23) **
Duration of overweight (years)	24 (19–37)	29 (20–42)	1.03 (0.99–1.07)
Unemployed (yes)	3 (7%)	10 (23%) *	4.22 (1.08–16.53) *

Data are shown as mean ± SEM, median (25th–75th percentile) or n (%), as appropriate. Odds ratios are calculated per unit or number, except for steps (per 100). For smoking, current and former smoker groups are combined. * *p* ≤ 0.05, ** *p* ≤ 0.01 for differences between groups or for odds ratio. ^#^ Never = 1/few portions a month = 2/one portion a week = 3/two to four portions a week = 4/five to six portions a week = 5/daily = 6. For fruits & vegetables, one portion comprised of e.g., fruit, small salad or side dish of vegetables. For red meat, one portion comprised of e.g., steak, roast beef or bolognaise. ^&^ Fatigue measured by Fatigue Impact Scale (0–160). ^##^ One portion was defined as a bottle (33cl) of beer, a glass (12cl) of medium alcohol wine, a glass (8cl) of high alcohol wine or a glass (4cl) of liquor. Abbreviations: E%, energy percent; EE, energy expenditure; MET, metabolic equivalent of task; MUFA, monounsaturated fatty acid; MVPA, moderate-to-vigorous physical activity; NAFLD, non-alcoholic fatty liver disease; OR, odds ratio; PA, physical activity; PUFA, polyunsaturated fatty acid; SFA, saturated fatty acid.

**Table 2:** Physical and biochemical characteristics, liver histology, and genetic characteristics of the participants without (FIB−) and with (FIB+) NAFLD-fibrosis, and univariable analysis to predict NAFLD-fibrosis.

	NAFLD-Fibrosis		OR (95% CI)
	No (FIB−, *n* = 48)	Yes (FIB+, *n* = 46)	
Physical characteristics			
Age (years)	49 (42–56)	53 (44–57)	1.03 (0.98–1.08)
Sex (female)	42 (88%)	32 (70%) *	0.29 (0.10–0.91) *
Weight (kg)	109 ± 3	116 ± 3	1.03 (0.98–1.08)
BMI (kg/m^2^)	39.1 ± 0.8	40.3 ± 0.7	1.04 (0.96–1.13)
Waist-to-hip ratio	0.91 ± 0.02	0.97 ± 0.02 *	1.06 (1.01–1.11) **
Systolic blood pressure (mmHg)	124 ± 2	125 ± 1.9	1.01 (0.98–1.04)
Diastolic blood pressure (mmHg)	79 (72–85)	77 (74–85)	1.00 (0.95–1.04)
Biochemical characteristics			
fP-glucose (mol/L)	5.7 ± 0.1	5.9 ± 0.1	1.41 (0.81–2.47)
HbA_1_c (mmol/mol)	35 (33–37)	38 (34–45) **	1.14 (1.04–1.24) **
fS-insulin (mU/L)	8.7 (6.2–10.8)	12.7 (7.6–17.9) ***	1.12 (1.04–1.21) **
HOMA-IR	2.2 (1.6–2.9)	4.2 (2.2–4.9) ***	1.56 (1.18–2.07) **
S-ALT (U/L)	24 (18–41)	30 (22–39)	1.01 (0.99–1.03)
S-AST (U/L)	25 (22–31)	27 (23–33)	1.03 (0.99–1.08)
AST/ALT-ratio	1.06 (0.72–1.29)	0.96 (0.81–1.13)	0.75 (0.34–1.64)
P-GGT (U/L)	21 (15–34)	28 (20–51) *	1.01 (1.00–1.03)
P-Albumin (g/L)	38.0 ± 0.3	38.9 ± 0.4	1.18 (0.99–1.40)
Platelet count (×10^9^/L)	259 (217–288)	260 (231–288)	1.00 (0.99–1.01)
fP-Triglycerides (mmol/L)	0.93 (0.80–1.20)	1.12 (0.87–1.42) *	4.62 (1.43–14.94) *
fP-HDL cholesterol (mmol/L)	1.21 (1.05–1.46)	1.13 (0.96–1.36)	0.27 (0.06–1.22)
fP-LDL cholesterol (mmol/L)	2.4 (2.1–3.1)	2.2 (1.9–2.9)	0.87 (0.57–1.31)
Lipid medication (yes)	11 (23%)	14 (30%)	1.47 (0.59–3.70)
Metabolic syndrome (yes)	23 (48%)	33 (72%) *	2.46 (1.04–5.80) *
Type 2 diabetes (yes)	12 (25%)	22 (48%) *	2.86 (1.17–7.00) *
Glucose-lowering medication (yes)	11 (23%)	20 (43%) *	2.59 (1.06–6.30) *
Liver histology			
Macrovesicular steatosis (%)	0 (0–5)	10 (0–20) ***	
Ballooning (0/1/2)	48/0/0	39/4/3 **	
Inflammation (0/1/2)	47/1/0	38/8/0 *	
Grade of activity (0/1/2/3)	47/1/0/0	38/1/4/3 *	
Stage of fibrosis (0/1/2/3/4)	48/0/0/0/0	0/39/6/1/0 ***	
Genetic characteristics			
*PNPLA3* (CC/CG/GG)	35/12/0	25/16/3 *	2.22 (0.91–5.34)
*TM6SF2* (CC/CT/TT)	40/7/0	37/7/0	0.93 (0.32–2.70)
*MBOAT7* (CC/CT/TT)	21/18/7	13/19/12	2.00 (0.85–4.57)
*HSD17B13* (--/-A/AA)	32/14/1	31/10/3	0.75 (0.36–2.11)
Genetic risk score (0/1/2/3/4/5/6/7/8)	0/4/17/14/11/0/0/0/0	0/3/8/11/15/6/1/0/0 **	1.79 (1.18–2.70) **

Data are shown as mean ± SEM, median (25th–75th percentile) or n (%), as appropriate. Odds ratios are calculated per unit or number, except for waist-to-hip ratio (per 0.01). * *p* ≤ 0.05, ** *p* ≤ 0.01, *** *p* ≤ 0.001 for differences between groups or for odds ratio. Abbreviations: ALT, alanine aminotransferase; AST, aspartate aminotransferase; BMI, body mass index; CI, confidence interval; FFA, free fatty acids; fP, fasting plasma; fS, fasting serum; GGT, γ-glutamyl-transferase; HbA_1C,_ glycosylated hemoglobin A1C; HDL, high-density lipoprotein; HOMA-IR, homeostasis model assessment of insulin resistance; *HSD17B1*, 17-β hydroxysteroid dehydrogenase; LDL, low-density lipoprotein; *MBOAT7*, membrane bound O-acyltransferase domain containing 7; NAFLD, non-alcoholic fatty liver disease; OR, odds ratio; P, plasma; *PNPLA3*, patatin-like phospholipase domain-containing protein 3; *TM6SF2*, transmembrane 6 superfamily member 2.

**Table 3:** Multivariable logistic regression models to predict NAFLD-fibrosis.

Model	AUROC (95% CI)	B	S.E.	OR (95% CI)	*p*-Value
‘Lifestyle model’	0.743 (0.643–0.843), *p* < 0.001				
Red meat intake ^#^		0.536	0.237	1.97 (1.18–3.28)	0.024
Carbohydrate E%		−0.098	0.042	0.91 (0.84–0.99)	0.020
Unemployment (no = 0/yes = 1)		2.033	0.826	7.63 (1.51–38.52)	0.014
Constant		1.950	2.015	0.008	0.994
‘Biomarker model’	0.776 (0.677–0.875), *p* < 0.001				
HbA_1c_ (mmol/mol)		0.102	0.048	1.11 (1.01–1.22)	0.032
Insulin (mU/L)		0.096	0.039	1.10 (1.02–1.19)	0.014
Triglycerides (mmol/L)		0.815	0.582	2.26 (0.72–7.07)	0.161
Constant		−5.931	1.777	0.003	0.001
‘Genetic model’	0.629 (0.513–0.745), *p* = 0.035				
Genetic risk score		0.465	0.203	1.59 (1.07–2.40)	0.022
Constant		−0.458	0.645	0.233	0.024
‘Biomarker + Genetic model’	0.824 (0.733–0.914), *p* < 0.001				
HbA_1c_ (mmol/mol)		0.126	0.051	1.13 (1.03–1.25)	0.014
Insulin (mU/L)		0.092	0.043	1.10 (1.01–1.19)	0.032
Triglycerides (mmol/L)		0.987	0.628	2.68 (0.78–9.19)	0.116
Genetic risk score		0.509	0.254	1.66 (1.01–2.74)	0.046
Constant		−8.424	2.273	0.000	0.000
‘Comprehensive model’	0.903 (0.841–0.964), *p* < 0.001				
MVPA (h/week)		−0.060	0.027	0.94 (0.89–0.99)	0.024
Red meat intake ^#^		0.581	0.303	1.79 (0.99–3.24)	0.055
Carbohydrate E%		−0.131	0.059	0.88 (0.78–0.99)	0.027
Smoking (never = 0/current or former = 1)		1.310	0.663	3.71 (1.01–13.60)	0.048
HbA_1c_ (mmol/mol)		0.186	0.067	1.21 (1.06–1.39)	0.006
Triglycerides (mmol/L)		2.504	0.981	12.23 (1.79–83.63)	0.011
Genetic risk score		0.936	0.333	2.55 (1.33–4.90)	0.005
Constant		−9.098	4.035	0.000	0.024

^#^ Never = 1/few times in a month = 2/once a week = 3/two to four times a week = 4/five to six times a week = 5/daily = 6. Abbreviations: AUROC, area under the receiver operating characteristic curve; B, coefficient; CI, confidence interval; E%, energy percentage; HbA_1c_, glycosylated hemoglobin A_1C_; MAQ, Modifiable Activity Questionnaire; OR, odds ratio; MVPA, moderate-to-vigorous physical activity, S.E., Standard Error.

## Data Availability

The data presented in this study are available on request from the corresponding author on reasonable request.
